# Supplementary material for: Evolution of cooperation in costly institutions exhibits Red Queen and Black Queen dynamics in heterogeneous public goods
Source: Commun Biol. 2021 Nov 29;4:1340. doi: 10.1038/s42003-021-02865-w (PMC8630072; doi:10.1038/s42003-021-02865-w)
Supplement: Supplementary file 2 — Supplementary Information [file 42003_2021_2865_MOESM2_ESM.pdf]

Supplementary Information for:
Evolution of Cooperation in Costly Institutions Exhibits Red Queen and Black Queen Dynamics in Heterogeneous Public Goods.

Mohammad Salahshour

Contents

Supplementary Note.1 Overview of the model **1**
Supplementary Note.1.1 Mixed population 1
Supplementary Note.1.2 Structured population 2
Supplementary Note.1.3 Exponential model 2
Supplementary Note.1.4 Continuous replicator dynamics 3
Supplementary Note.1.5 Replicator dynamics for the exponential model 3

Supplementary Note.2 Density of different strategies and comparison with simulations in the well-mixed population **4**

Supplementary Note.3 Dependence on the mutation rate **6**
Supplementary Note.3.1 Non-zero mutation rate 6
Supplementary Note.3.2 Zero mutation rate (pure selection) 6

Supplementary Note.4 Continuous replicator dynamics **7**

Supplementary Note.5 Interaction between two costly institutions **7**

Supplementary Note.6 Structured population **8**

Supplementary Note.7 Supplementary videos **8**

Supplementary Note.8 The exponential model **9**

Supplementary Note.1 Overview of the model

We consider both a mixed population and a structured population. Below we overview the model for these cases separately.

Supplementary Note.1.1 Mixed population

For the case of a mixed population, we consider a population of N individuals. At each time step, groups of g individuals are drawn randomly from the population pool to play the game. Individuals in each group can choose between two public resources, resource 1 and resource 2. Resource 1 is a costly institution. Entrance to this public resource requires individuals to pay an entrance or participation fee. Resource 2, on the other hand, is a free institution and requires no entrance fee. Individuals gather payoff by playing a public goods game in their institution. In this game, individuals can either

cooperate or defect. Cooperators pay a cost c to invest the same amount in the public resource. Defectors pay no cost and do not invest. All the investments in a public resource i are multiplied by an enhancement factor r_i and are divided equally among the individuals in that resource. In addition to the public goods game, we assume individuals receive a base payoff, b, from other activities not related to the public goods game.

After playing the games, individuals reproduce with a probability proportional to their payoff. In the reproduction stage, the whole population is updated such that the population size remains constant. That is, for each individual in the next generation, an individual is chosen as a parent with a probability proportional to its payoff. The offspring inherit the game preference (which can be institution 1 or 2) and the game strategy (which can be cooperation C or defection D) of its parent, subject to mutations. Mutations in the game preference and strategy occur independently, each with probability ν. In the case of a mutation, the corresponding variable's value is changed to its opposite value (for example, 1 to 2 for the game preference and C to D for the game strategy).

Supplementary Note.1.2 Structured population

In the case of a structured population, we consider a population of individuals residing on a network. The population network is taken to be a first nearest neighbor square lattice with von Neumann connectivity and periodic boundaries. Each individual participates in five groups, each centered around itself or one of its neighbors, to perform a collective action task. Individuals in each group enter their preferred institution and play a public goods game in their preferred institution. In addition to the payoff from the public goods games, individuals receive a base payoff of b. After deriving their payoffs, the whole population is updated. For reproduction, we consider an imitation dynamics in which each individual imitates the strategy of one of the individuals in its extended neighborhood chosen with a probability proportional to its payoff. The extended neighborhood of an individual is composed of the individual and its neighbors. We assume mutations can occur as well. After imitation, the strategy and the game preference of the individual mutate independently and each with probability ν.

Supplementary Note.1.3 Exponential model

In Fig. 5 in the main text and in the following in this text, we also consider a second model in which individuals reproduce with a probability proportional to the exponential of their payoff. The only modification of this model, which we call the exponential model, is that in the reproduction stage, instead of reproducing with a probability proportional to their payoff, individuals reproduce with a probability proportional to the exponential of their payoff times a selection parameter β. That is, the fitness of an individual i, with payoff π_i is defined as $\exp(\beta\pi_i)$, and the individuals are drawn according to their fitness to produce offspring. All the other details of the model remain as before. The replicator dynamics for this model are derived in the next section.

In the case of a structured population, in the exponential model, each individual imitates the strategy of an individual in its extended neighborhood with a probability proportional to $\exp(\beta\pi_i)$, where β is a selection parameter, and π_i is the payoff of an individual i in the extended neighborhood of the focal individual. All the other aspects of the model remain as before.

Supplementary Note.1.4 Continuous replicator dynamics

The continuous replicator-mutator dynamics can be written as follows:

$$\frac{d\rho_x^i}{dt} = \sum_{y,j} \nu_{y,j}^{x,i} \rho_y^j(t) \pi_y^j(t) - \sum_{z,l} \rho_z^l(t) \pi_z^l(t). \qquad \text{(Supplementary Equation.1)}$$

Here, x, y, and z refer to strategies and can be either C or D, and i, j, and l refer to the public resources which can be 1 or 2. $\nu_{y,j}^{x,i}$ is the mutation rate from a strategy profile that prefers public resource j and plays strategy y to a strategy profile that prefers public resource i and plays strategy x. These can be written in terms of mutation rates as follows:

$$\begin{cases} \nu_{y,j}^{x,i} = 1 - 2\nu + \nu^2, & \textit{if} \quad (i = j \quad \textit{and} \quad x = y) \\ \nu_{y,j}^{x,i} = \nu - \nu^2, & \textit{if} \quad (i = j \quad \textit{and} \quad x \neq y) \quad \textit{or} \quad (i \neq j \quad \textit{and} \quad x = y) \\ \nu_{y,j}^{x,i} = \nu^2 & \textit{if} \quad (i \neq j \quad \textit{and} \quad x \neq y) \end{cases}$$

(Supplementary Equation.2)

In Supplementary Equation.1, π_y^j is the expected payoff of an individual who prefers public resource j and plays strategy y and is calculated in the Methods Section in the main texts. Using expressions in eq. 3 in the main text for payoffs in Supplementary Equation.1, we arrive at the continuous replicator-mutator dynamics for the model.

Supplementary Note.1.5 Replicator dynamics for the exponential model

The replicator dynamics for the model considered in the main text, in which individuals reproduce with a probability proportional to their payoff, is derived in the main text. In this section, we derive the replicator dynamics for the exponential model, in which individuals reproduce with a probability proportional to the exponential of their payoff. The argument follows similar steps to those developed in the Methods section in the main text. For completeness, we detail the derivation here.

We begin by writing the replicator dynamics of the model, which reads as follows:

$$\rho_x^i(t+1) = \sum_{y,j} \nu_{y,j}^{x,i} \rho_y^j(t) \frac{w_y^j(t)}{\sum_{z,l} \rho_z^l(t) w_z^l(t)}. \qquad \text{(Supplementary Equation.3)}$$

x, y, and z refer to strategies and can be either C or D, and i, j, and l refer to the public resources which can be 1 or 2. $\nu_{y,j}^{x,i}$ is the mutation rate from a strategy profile that prefers public resource j and plays strategy y to a strategy combination that prefers public resource i and plays strategy x. These can be written in terms of the mutation rate as in Supplementary Equation.2. In Supplementary Equation.3, w_y^j is the expected fitness of an individual who prefers public resource j and plays strategy y. These terms can be written by averaging a focal individual's fitness with game preference j (for $j = 1$ and 2) in a group composed of n_C^j cooperators and n_D^j defectors who prefer public resource j,

over all possible group configurations. In this way, we have the following equations for the fitness:

$$w_C^1 = \sum_{n_D^1=0}^{g-1-n_C^1} \sum_{n_C^1=0}^{g-1} \exp\left[\beta(cr_1 \frac{1+n_C^1}{1+n_C^1+n_D^1} - c - c_g + \pi_0)\right]$$
$$(1-\rho_C^1-\rho_D^1)^{g-1-n_C^1-n_D^1} {\rho_D^1}^{n_D^1} {\rho_C^1}^{n_C^1} \binom{g-1}{n_C^1, n_D^1, g-1-n_C^1-n_D^1},$$
$$w_D^1 = \sum_{n_D^1=0}^{g-1-n_C^1} \sum_{n_C^1=0}^{g-1} \exp\left[\beta(cr_1 \frac{n_C^1}{1+n_C^1+n_D^1} - c_g + \pi_0)\right]$$
$$(1-\rho_C^1-\rho_D^1)^{g-1-n_C^1-n_D^1} {\rho_D^1}^{n_D^1} {\rho_C^1}^{n_C^1} \binom{g-1}{n_C^1, n_D^1, g-1-n_C^1-n_D^1}.$$

$$w_C^2 = \sum_{n_D^2=0}^{g-1-n_C^2} \sum_{n_C^2=0}^{g-1} \exp\left[\beta(cr_2 \frac{1+n_C^2}{1+n_C^2+n_D^2} - c + \pi_0)\right]$$
$$(1-\rho_C^2-\rho_D^2)^{g-1-n_C^2-n_D^2} {\rho_D^2}^{n_D^2} {\rho_C^2}^{n_C^2} \binom{g-1}{n_C^2, n_D^2, g-1-n_C^2-n_D^2},$$
$$w_D^2 = \sum_{n_D^2=0}^{g-1-n_C^2} \sum_{n_C^2=0}^{g-1} \exp\left[\beta(cr_2 \frac{n_C^2}{1+n_C^2+n_D^2} + \pi_0)\right]$$
$$(1-\rho_C^2-\rho_D^2)^{g-1-n_C^2-n_D^2} {\rho_D^2}^{n_D^2} {\rho_C^2}^{n_C^2} \binom{g-1}{n_C^2, n_D^2, g-1-n_C^2-n_D^2}. \quad \text{(Supplementary Equation.4)}$$

In this equation, $\exp\left[\beta(cr_1 \frac{1+n_C^1}{1+n_C^1+n_D^1} - c - c_g + \pi_0)\right]$ in the first equation is the fitness of a cooperator who prefers the public resource 1, and $\exp\left[\beta(cr_1 \frac{n_C^1}{1+n_C^1+n_D^1} - c_g + \pi_0)\right]$ in the second equation is the fitness of a defector who prefers public resource 1. $\binom{g-1}{n_c^1, n_D^1, g-1-n_C^1-n_D^1} = \frac{(g-1)!}{n_C^1! n_D^1! (g-1-n_C^1-n_D^1)!}$ is the multinomial coefficient and is the number of ways that n_C^1 cooperators and n_D^1 defectors who prefer game 1 can be chosen among $g-1$ group-mates of a focal individual. $(1-\rho_C^1-\rho_D^1)^{g-1-n_C^1-n_D^1} {\rho_D^1}^{n_D^1} {\rho_C^j}^{n_C^1} \binom{g-1}{n_c^1, n_D^1, g-1-n_C^1-n_D^1}$, is the probability that such a group composition occurs. Summation over all the possible configurations gives the expected payoff of a cooperator, or defector who prefers resource 1.

It is easy to derive the expected fitness of those who prefer public resources 2 by using a similar argument. In our analysis of the exponential model, we set $\pi_0 = 0$.

Supplementary Note.2 Density of different strategies and comparison with simulations in the well-mixed population

The density of different strategies, resulted from the replicator dynamics (top panels) and a simulation in a population of $N = 10000$ individuals (bottom), are plotted in Supplementary Figure.1, Supplementary Figure.2, and Supplementary Figure.3. Here the same parameter values used in the main text are used, namely, $g = 5$ and $\nu = 0.001$. The replicator dynamics are solved for 8000 time

steps starting from a uniform initial condition in which all the strategies' densities are equal. An average over the last 2000 time steps is taken. The simulations are performed for 6000 time steps starting from a uniform initial condition in which the individuals' strategies are randomly assigned. An average over the last 3000 time steps is taken. Supplementary Figure.1 shows the density of different strategies in the $c_g - r$ plane. Here, $r_1 = r_2 = r$. Supplementary Figure.2 and Supplementary Figure.3 show the densities of strategies in $r_1 - r_2$ plane, for respectively, $c_g = 0.1$ and $c_g = 0.4$. As can be seen, the replicator dynamics results, an exact solution of the model in the infinite population limit, is in high agreement with the simulation results in a finite population.

In the $c_g - r$ plane (Supplementary Figure.1), the dynamics settles in a fixed point for both small and large enhancement factors. In between, in the region marked by dashed white lines, the dynamics show periodic fluctuations. The periodic orbit can be decomposed into a red queen orbit for small enhancement factors, in which cooperation only in the costly institution evolves, and a black queen orbit for large enhancement factors in which cooperation in both the costly and free institutions evolve. For small costs, the system also shows a bistable region (between the green circles and red squares) where both periodic orbits (or the black queen orbit and a fixed point to the right of the dashed white line) are stable.

Similar phases exist in the $r_1 - r_2$ planes. For small costs, as in Supplementary Figure.2, the system settles in a fixed point for both small and large enhancement factors, and the black queen periodic orbit occurs in between. In this case, the system also shows a bistable region for medium enhancement factors where both a fixed point and a periodic orbit are possible. On the other hand, for high costs, as in Supplementary Figure.3, the system shows no bistability. This shows that a large entrance cost destabilizes full defection and removes the bistability of the system. Consequently, starting from all the initial conditions, cooperators survive in the system either in a fixed point (outside the dashed white line) or a periodic orbit (inside the dashed white line). Besides, we note that a large entrance cost not only promotes cooperation in the costly institution but also by removing bistability in medium enhancement factors ensures the evolution of cooperation in the free institution. In addition, for a high cost, the system shows a markedly higher cooperation level for small enhancement factors. The red queen periodic orbit, in which cooperation evolves only in the costly institution, occurs for small r_2, and the black queen periodic orbit, where cooperation in both institutions evolves, occurs for larger values of r_2.

We also plot the time average densities of different strategies as a function of $r_1 = r_2 = r$, for three different values of c_g, in Supplementary Figure.4. Blue dots and red squares represent the solutions of the replicator dynamics for two different initial conditions, and orange circles show the results of simulations starting from a uniform initial condition in which the individuals' strategies are assigned at random. The bistability of the dynamics for small costs can be seen in Supplementary Figure.4 (top panels). Here, the blue dots and red squares show the replicator dynamics' stationary state starting from two different initial conditions. While for both small and large r the dynamic is mono-stable and the attractors of the dynamic for different initial conditions coincide, the situation is different for medium values of r: For r between around 2 and 3, the system shows a bistable region where the two initial conditions result in different attractors. On the other hand, for large costs (bottom panels), no bistability is observed, and the stationary state of the dynamics is independent of the initial condition. In this region, by increasing r, the system shows a cross-over from the defective periodic orbit to the cooperative periodic orbit without passing any singularity. In between, the transition between the two periodic orbits becomes continuous at a single critical point (middle panel).

Supplementary Note.3 Dependence on the mutation rate

Supplementary Note.3.1 Non-zero mutation rate

To see the dependence of the results on the mutation rate, in Supplementary Figure.5, we present the densities of different strategies in the $c_g - r$ plane for two different mutation rates. Here, the replicator dynamics is solved starting from a uniform initial condition, setting the mutation rate equal to 10^{-4} (top panels) and $\nu = 10^{-2}$ (bottom panel). The phase diagram of the model is superimposed as well. Comparison of the result for $\nu = 10^{-4}$ with that for $\nu = 10^{-3}$ in Supplementary Figure.1 shows that the size of the region of the phase diagram where the dynamics settle in a periodic orbit increases for lower mutation rate. For a larger mutation rate, $\nu = 10^{-2}$, on the other hand, no periodic orbit is observed in the $c_g - r$ plane. Instead, the dynamics settle in a fixed point in the entire phase diagram. Besides, no bistability is observed for larger mutation rates. Instead, the dynamics show a cross-over from a state with a low level of cooperation to a state with a high level of cooperation by increasing r. In addition, the density of cooperators in the free institution decreases for lower mutation rates. On the other hand, the density of cooperators in the costly institution slightly increases for large enhancement factors, for larger mutation rates.

Supplementary Note.3.2 Zero mutation rate (pure selection)

We also consider the case of zero mutation rate, where the dynamic is driven purely by selection. We plot the density of different strategies resulting from the replicator dynamics with zero mutation rate in Supplementary Figure.6. Here the replicator dynamic is solved starting from a uniform initial condition in which all the strategies' initial densities are equal. As for the non-zero and low mutation rates, the dynamics settle in a fixed point or periodic orbits. However, in this case, in a fixed point, only one of the strategies survives and dominates the population. An exception occurs for $c_g = 0$ where either defectors (for small enhancement factors) or cooperators (for large enhancement factors) in both institutions survive and coexist in the same frequencies. For non-zero c_g, however, non-costly defectors dominate for small enhancement factors. For larger enhancement factors and medium to high costs, costly cooperators dominate and drive other strategies to extinction. Finally, for large enhancement factors, non-costly cooperators dominate the population. In addition to the fixed points, the dynamics show two different periodic orbits. A red queen periodic orbit where costly cooperators survive and coexist with costly defectors and non-costly defectors occurs for small enhancement factors. On the other hand, for too large enhancement factors, yet smaller than $g = 5$, the black queen periodic orbit where costly cooperators, non-costly cooperators, and non-costly defectors survive occurs. In contrast to the non-zero mutation rate, where costly defectors may also appear in the black queen orbit, with zero mutation rate, defection in the costly institution is more efficiently suppressed.

Examples of the red queen and black queen periodic orbits for zero mutation rate are presented in Supplementary Figure.7. In this case, a similar phenomenology to that observed for the non-zero mutation rate is at work. Namely, in the red queen periodic orbit (Supplementary Figure.7(a) to Supplementary Figure.7(c)) costly cooperators, costly defectors and non-costly defectors survive. Furthermore, despite the evolution of cooperation, the average payoff remains close to zero, and the payoff of costly cooperators and costly defectors fluctuates around zero. However, compared to the non-zero mutation rate, both the amplitude and the period of fluctuations increase, and the dynamics of the system go through periods where one of the strategies almost dominates the entire population. This is also the case for the black queen periodic orbit (Supplementary Figure.7(d) to (f)). In this case, non-costly cooperators often dominate the population. However, the dynamics go through outbursts of defection during which non-costly defectors take over the population. At these stages, costly cooperators, although in disadvantage in the presence of non-costly cooperators, receive a higher

payoff compared to non-costly defectors (whose payoff becomes close to zero once the frequency of non-costly cooperators drops to a value close to zero) and replace the latter. The dominance of costly cooperators, in turn, gives rise to the evolution of costly defectors who can exploit costly cooperators and replace them. At this stage, non-costly cooperators, immune to costly defectors, reemerge in high frequency. In this way, the dynamics of the system go through rock-paper-scissors dynamics in which non-costly cooperators, non-costly defectors, and costly cooperators alternately dominate the population.

Supplementary Note.4 Continuous replicator dynamics

We also examine the dynamics of the model under continuous replicator dynamics. Continuous replicator dynamics show similar behavior to discrete replicator dynamics. This can be seen in Supplementary Figure.8, where the density of different strategies is plotted in the $c_g - r$ plane. Similar phases to the discrete replicators dynamics are observed, namely, a defective fixed point and a cooperative fixed point for, respectively, small and large enhancement factors. In between the systems shows periodic orbits. In the periodic phase, for smaller enhancement factors, a red queen orbit where cooperation only in the costly institution evolves occurs. For higher enhancement factors, a black queen orbit where cooperation in both the costly and free institution evolves occur. We note that, as we will see in the next section, the size of the periodic region increases with decreasing mutation rate. Compared to the discrete replicator dynamics, for the same mutation rate, the size of the periodic region decreases under the continuous replicators dynamics. For instance, for the base mutation rate of $\nu = 10^{-3}$ the continuous replicator dynamics do not show periodic orbits. However, for lower mutation rates (as in Supplementary Figure.8 where $\nu = 10^{-8}$), periodic orbits are observed under the continuous replicator dynamics, and the size of the periodic phase increases by decreasing the mutation rate.

Examples of the two types of periodic orbits, red queen and black queen orbits, under the continuous replicator dynamics are presented in Supplementary Figure.9. As was the case in the discrete replicator dynamics, cooperation only in the costly institution evolves in the red queen periodic orbit (Supplementary Figure.9(a) and Supplementary Figure.9(b)). In this orbit, costly cooperators, costly defectors, and non-costly defectors coexist, and their frequency in the population shows periodic fluctuations. However, the payoff of costly cooperators and costly defectors from the game fluctuates around zero, and none is able to do better than non-costly defectors. In the black queen periodic orbit on the other hand, cooperation in both institutions evolve (Supplementary Figure.9(c) and Supplementary Figure.9(d)). In this orbit, costly cooperators, non-costly cooperators, and non-costly defectors coexist and show periodic fluctuations. Defection in the costly institution, on the other hand, remains at a low level.

Supplementary Note.5 Interaction between two costly institutions

The phenomenology of the model is similar when instead of a costly institution and a cost-free institution, two costly institutions coexist. To see this, assume institution 1 has an entrance cost c_g, and institution 2 has an entrance cost c_g^0. Without loss of generality assume $c_g > c_g^0$. In such a case, all the individuals need to pay a cost c_g^0. Those who choose the costlier resource have to pay an additional cost of $c_g - c_g^0$. As all the individuals pay a cost, c_g^0, c_g^0 can be absorbed to the base payoff, b. Thus this model is equivalent to a model where costly and cost-free institutions coexist, but with a shifted base payoff, $b - c_g^0$. This argument can also be easily seen from the replicator dynamics by absorbing the term c_g^0 into the base payoff b in the equation for those who prefer public resource 2, ρ_C^2, and ρ_D^2.

Similarly, by writing $c_g = c_g^0 + (c_g - c_g^0)$ in the equations for rho_C^1 and ρ_D^1, it is possible to absorb the term c_g^0 into the base payoff.

In Supplementary Figure.10, we consider such a case. Here, we have set $c_g^0 = 0.1$, and color plotted the frequency of different strategies as a function of $c_g - c_g^0$. The phase diagram of the model is superimposed as well. As can be seen, a similar phase diagram and similar frequencies of different strategies are observed in this case.

Supplementary Note.6 Structured population

To better compare the model's behavior in the structured population with that in the mixed population, in Supplementary Figure.11 we present the time average densities of different strategies in a structured population for two different values of the participation cost, as a function of r. Here, a population of 160000 individuals residing on an 800×800 square lattice with von Neumann connectivity and periodic boundaries is considered. As can be seen, for small enhancement factors, only non-costly defectors survive. As the enhancement factors increase, costly cooperators, but not non-costly cooperators, start to appear in the population. The density of costly cooperators experiences a peak for a moderate value of enhancement factor. Beyond this point, non-costly cooperators increase in density and form the majority of the population.

To look at the model's behavior in a structured population when the two resources have different qualities, in Supplementary Figure.12 we plot the densities of different strategies in the $r_1 - r_2$ plane for two different participation costs. In the top panels $c_g = 0.1$, and in the bottom panels $c_g = 0.4$. As mentioned before, contrary to a mixed population, the model does not show bistability in a structured population. Consequently, the behavior of the model is qualitatively similar for small and large costs. For too small r_1, for all the values of r_2, only non-costly defectors, D^2 survive. On the other hand, for r_1 larger than a small value (approximately $r_1 = 1.5$) costly cooperators survive even for r_2 equal or smaller than 1. This shows, similarly to the case of a mixed population, having a choice between different resources is not necessary to promote cooperation in a costly institute. Rather, cooperation evolves in a costly institute even for relatively small enhancement factors as long as participation is optional. Increasing r_2 however increases cooperation level in the costly institute (as long as $r_2 < r_1$). This results from the beneficial effect of freedom of choice between different public resources for the evolution of cooperation. Comparing the results for a small and a high participation cost shows that higher participation cost improves cooperation level in the costly institute for small r_2, i.e., when a viable alternative does not exist.

Supplementary Note.7 Supplementary videos

In the Supplementary Videos (SV) (available at https://doi.org/10.6084/m9.figshare.16712806.v1, Figshare), we present examples of the system's time evolution in different regimes. In the videos, a population of size 40000 individuals resides on a 200 square lattice with von Neumann connectivity and periodic boundaries. All the simulations start from a random assignment of the strategies.

In SV.1 to SV.4, the exponential model is used. In all the cases $c_g = 0.6$. In SV.1, $r_1 = r_2 = 1.7$. This phase corresponds to the red queen osculations. As can be seen in the video, in this regime, small domains of costly cooperators survive in a sea of non-costly defectors. Both non-costly cooperators and costly defectors can survive only in the neighborhood of the costly cooperators. By benefiting from a higher payoff in the neighborhood of costly cooperators, the two former types can expand at the expense of shrinking the domain of costly cooperators. However, they lose advantage once costly cooperators are out of the picture and shrink in the sea of non-costly defectors. The resulting

dynamics give rise to blocks of costly cooperators moving in the sea of non-costly defectors, followed by small bands of both costly defectors and non-costly cooperators.

In SV.2, $r_1 = r_2 = 2.2$. This phase corresponds to the black queen dynamics. The system shows strong spatiotemporal oscillations in which costly cooperators and non-costly defectors, as well as non-costly defectors, coexist. Costly defectors, however, exist only in significantly smaller frequencies. The spatiotemporal fluctuations go through three cycles in which non-costly defectors grow by replacing non-costly cooperators. Costly cooperators, in turn, grow in the domains of non-costly defectors. Lastly, non-costly cooperators reemerge once non-costly defectors are eliminated by costly cooperators.

In SV. 3 $r_1 = r_2 = 3.5$. In this regime, non-costly cooperators dominate the population. Non-costly defectors can survive by forming small bands in the sea of non-costly cooperators. Costly cooperators, while at a disadvantage in the sea of non-costly cooperators, experience advantage in the bands of non-costly defectors and can grow in their domains. Consequently, in the course of evolution, costly cooperators help non-costly cooperators by moving within the bands of non-costly defectors and purging the population out of non-costly defectors.

In SV. 4 the regime of $r_1 > r_2$ is considered. Here, $r_1 = 3$ and $r_2 = 1.8$. In this regime, costly cooperators compose the majority of the population. Costly defectors can survive in small islands in the sea of costly cooperators. Both non-costly cooperators and non-costly defectors, while at a disadvantage in the sea of costly cooperators, can grow in the islands of costly defectors, and thus, help the evolution of costly cooperators.

In SV. 5, the model in which individuals reproduce with a probability proportional to their payoff is considered. In this video, $c_g = 0.6$, $r_1 = r_2 = 1.7$. This phase corresponds to SV.1 for the exponential model. As can be seen in the video, a similar dynamic to that observed in the exponential model is observed here. This is also the case for different regimes, and the two models give rise to qualitatively similar dynamics.

Supplementary Note.8 The exponential model

This section presents results for a model where individuals reproduce with a probability proportional to the exponential of their payoff.

We begin with a mixed population. The color plot of different strategies in the $c_g - r$ plane is presented in Supplementary Figure.13. Here the phase diagram of the model resulting from the replicator dynamics is superimposed on the figures as well. The top panels show numerical solutions of the replicator dynamics, and the bottom panels show the simulation results. In Supplementary Figure.14, the density of different strategies for a fixed participation cost, $c_g = 0.4$ are color plotted in the $r_1 - r_2$ plane. The top panels show the replicator dynamics results, and the bottom panels show the results of simulations. The white lines show the boundary of the cyclic phase. Comparison with the results of the model in which individuals reproduce with a probability proportional to their payoff shows similar phases, and a similar phenomenology exists in both cases.

The results of simulations in a structured population in the exponential model are presented in Supplementary Figure.15 and Supplementary Figure.16. In Supplementary Figure.15 the density of different strategies in the $c_g - r$ plane is plotted and in Supplementary Figure.16 the density of different strategies in the $r_1 - r_2$ plane is plotted. In this case, a similar phenomenology to that observed in the model considered in the main text is observed too. This argues that our results are rather robust with respect to different update rules and variations of the model.

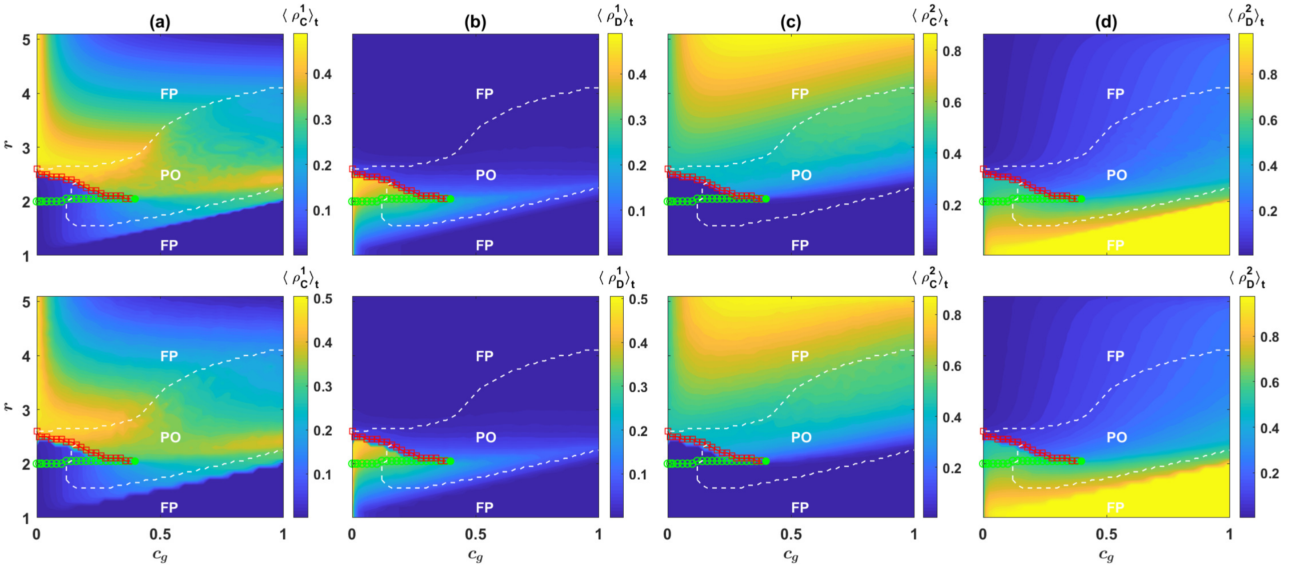

Supplementary Figure.1: The density of different strategies in the $c_g - r$ plane resulting from the replicator dynamics and simulations. The densities of different strategies in the $c_g - r$ plane are color plotted. The top panel shows the results of the replicator dynamics, and the bottom panel shows the results of simulations. The phase diagram of the model, derived from the replicator dynamics, is superimposed. For both small and large enhancement factors, r, the dynamics settle in a fixed point, denoted by FP. In between, in the region between the white lines denoted by PO, the dynamics settle in periodic orbits. The periodic orbit can be a red queen orbit (for smaller enhancement factors) where cooperation evolves only in the costly institute, or a black queen orbit (for larger enhancement factors), where cooperation in both institutes evolves. For a small cost, the model is bistable for medium values of r. Green circles show the lower boundary of the bistable region, above which the black queen periodic orbit becomes stable. The red squares show the upper boundary of the bistable region above which the dynamics settle in the black queen periodic orbit starting from all the initial conditions. The filled green circle shows the point where the transition between the two periodic orbits becomes a continuous transition. Parameter values: $g = 5$, $\nu = 10^{-3}$, and $\pi_0 = 2$. The replicator dynamic is solved for 8000 time steps, and the time averages are taken over the last 2000 steps. The simulation is performed in a population of size $N = 10000$ individuals for 6000 time steps. An average over the last 3000 time steps is taken. The initial condition is a random assignment of the strategies.

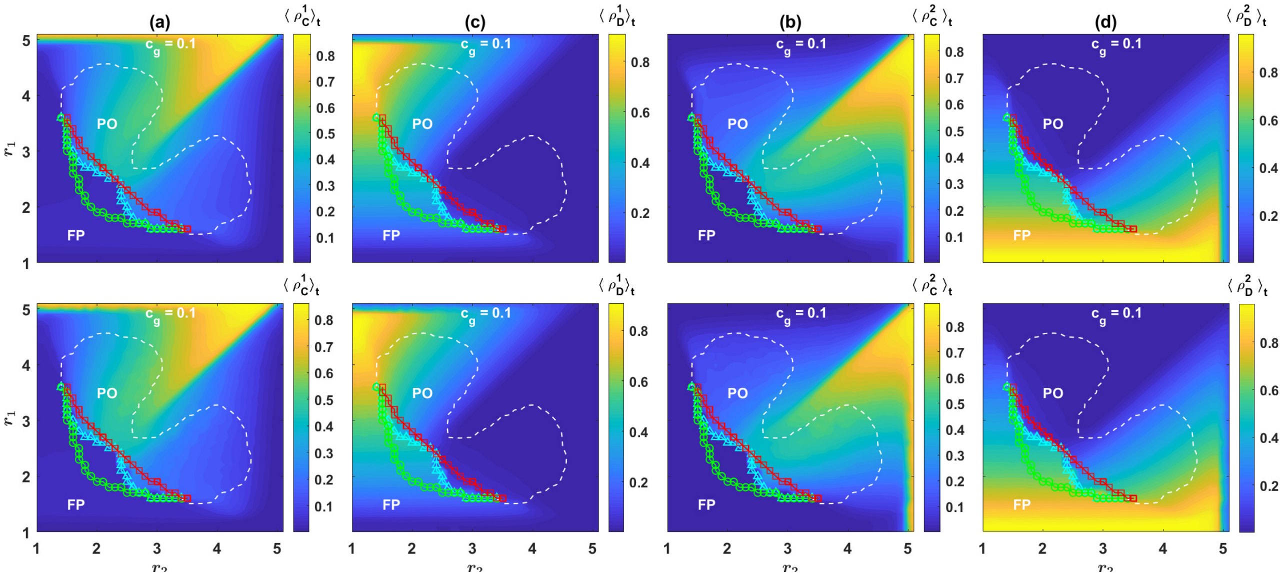

Supplementary Figure.2: The density of different strategies in the $r_1 - r_2$ plane resulting from the replicator dynamics and simulations, for $c_g = 0.1$. The densities of different strategies in the $r_1 - r_2$ plane, resulting from the replicator dynamics (top) and simulations (bottom), are color plotted. The phase diagram of the model, derived from the replicator dynamics, is superimposed. The dynamics settle in a fixed point for both too small and too large enhancement factors. The black queen periodic orbit occurs in between. The white dashed line shows the boundary of the periodic phase. For medium enhancement factors, the dynamics show bistability. Green circles show the lower boundary of the bistable region, above which the black queen periodic orbit becomes stable. The red squares show the upper boundary of the bistable region above which the dynamics settle in the black queen periodic orbit starting from all the initial conditions. Blue triangles show the phase boundary, resulting from a uniform initial condition. Parameter values: $g = 5$, $\nu = 10^{-3}$, and $\pi_0 = 2$. The replicator dynamic is solved for 8000 time steps, and the time averages are taken over the last 2000 steps. The simulation is performed for 6000 time steps, and an average over the last 3000 time steps is taken. The initial condition is a uniform initial condition.

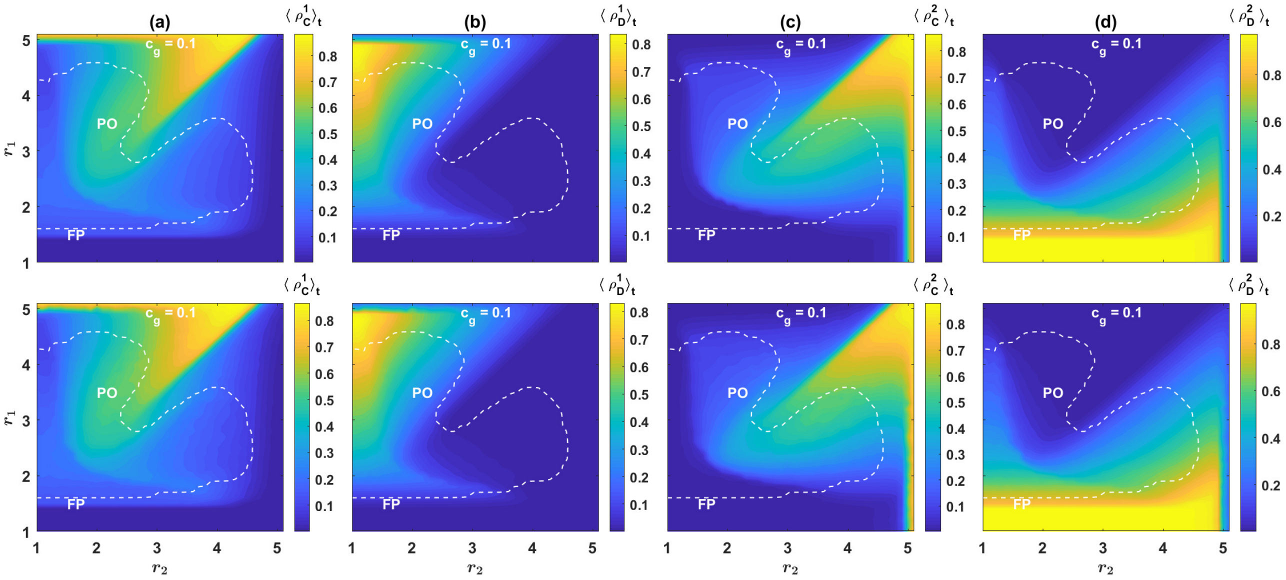

Supplementary Figure.3: The density of different strategies in the $r_1 - r_2$ plane resulting from the replicator dynamics and simulations, for $c_g = 0.4$. The densities of different strategies in the $r_1 - r_2$ plane, resulting from the replicator dynamics (top) and simulations (bottom), are color plotted. The phase diagram of the model, derived from the replicator dynamics, is superimposed. The dynamics settle in a fixed point for both too small and too large enhancement factors. Periodic fluctuations occur in between. The white dashed line shows the boundary of the cyclic phase. The cyclic phase is composed of a black queen periodic orbit (for larger enhancement factors), in which cooperation in both institutions evolves, and a red queen periodic orbit (for smaller enhancement factors), in which cooperation only in the costly institution evolves. Parameter values: $g = 5$, $\nu = 10^{-3}$, and $\pi_0 = 2$. The replicator dynamic is solved for 8000 time steps, and time averages are taken over the last 2000 steps. The simulation is performed for 6000 time steps, and an average over the last 3000 time steps is taken. The initial condition is a uniform initial condition.

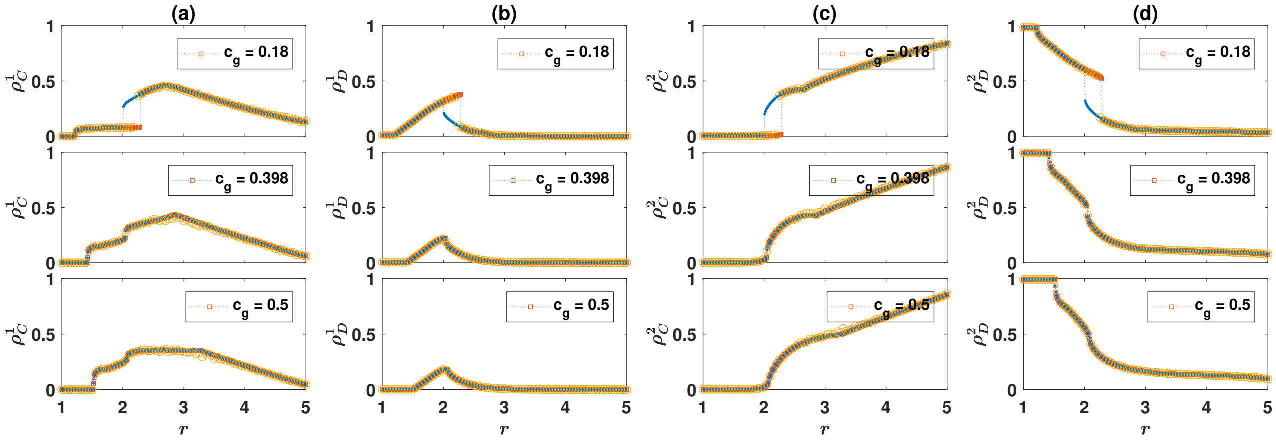

Supplementary Figure.4: Density of different strategies as a function of r for three different values of cost. The replicator dynamics is solved for two different initial conditions, a cooperation favoring initial condition in which all the individuals are cooperators and prefer the costly institution (blue dots), and a uniform initial condition in which strategy and game preference of the individuals are assigned at random (red squares). The result of simulations in a population of size $N = 10000$ starting from a random initial condition is shown by orange circles. The system shows two different cooperative phases. For small enhancement factors, cooperation in the costly institution, but not in the free institution, evolves. For larger enhancement factors, cooperation in both costly and free institutions evolves. While for a small cost, the transition between the two cooperative phases is discontinuous and shows bistability (a), for high cost, there is a cross-over between the two phases by increasing the enhancement factor (c). Parameter values: $g = 5$, $\nu = 10^{-3}$, $\pi_0 = 2$. The replicator dynamic is solved for 9000 time steps, and the time averages are taken over the last 2000 time steps. The simulation is performed for 6000 time steps, and the averages are taken for the last 3000 time steps.

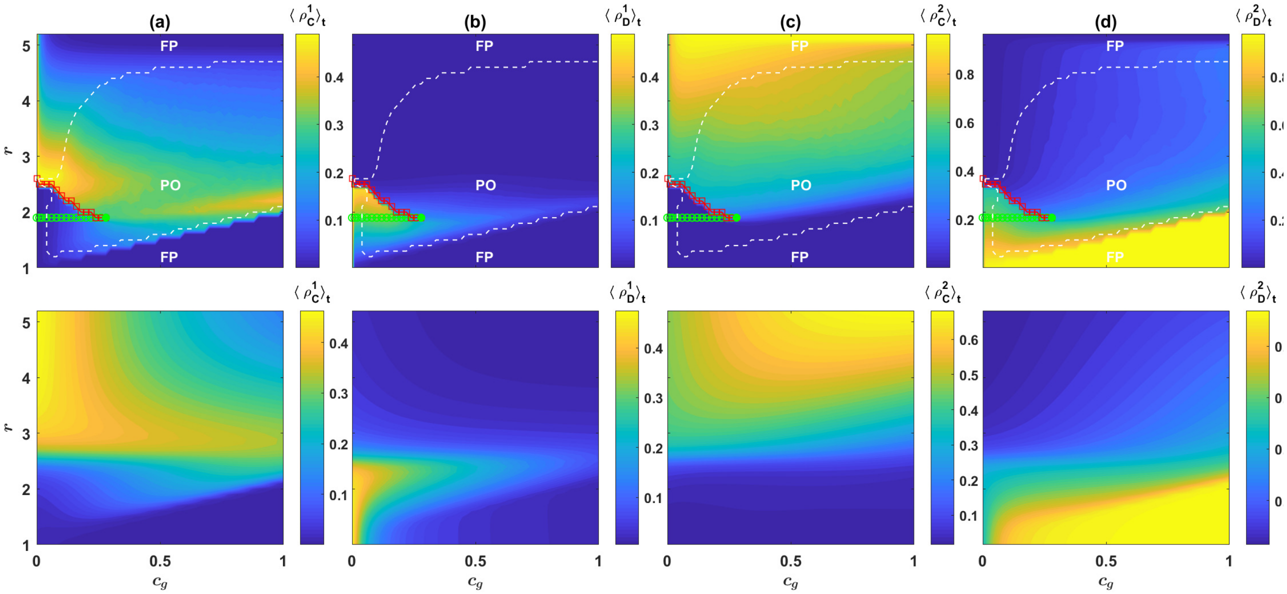

Supplementary Figure.5: The density of different strategies in the $c_g - r$ plane for two different mutation rates. The densities of different strategies in the $c_g - r$ plane are color plotted for two different mutation rates. The phase diagram of the model is superimposed. In the top panels $\nu = 10^{-4}$, and in the bottom panels $\nu = 10^{-2}$. For small mutation rate (top), the model can settle in a fixed point (FP) or periodic orbits (PO) and shows bistability for small costs. White lines show the boundary of the cyclic phases. The periodic orbit is a red queen orbit, in which cooperation only in the costly institute evolves (for small enhancement factors), or a black queen orbit, in which cooperation in both institutions evolves (for large enhancement factors). Green circles show the lower boundary of the bistable region, above which the black queen periodic orbit becomes stable, and the red squares show the upper boundary of the bistable region above which the dynamics settle in the black queen periodic orbit starting from all the initial conditions. The filled green circle shows the critical point, where the transition between the two periodic orbits becomes a continuous transition. For a large mutation rate (bottom), the model settles in a fixed point in the entire $c_g - r$ plane, and no bistability is observed. Here, $\pi_0 = 2$ and $g = 5$. The replicator dynamic is solved for 8000 time steps, and time averages are taken over the last 2000 steps.

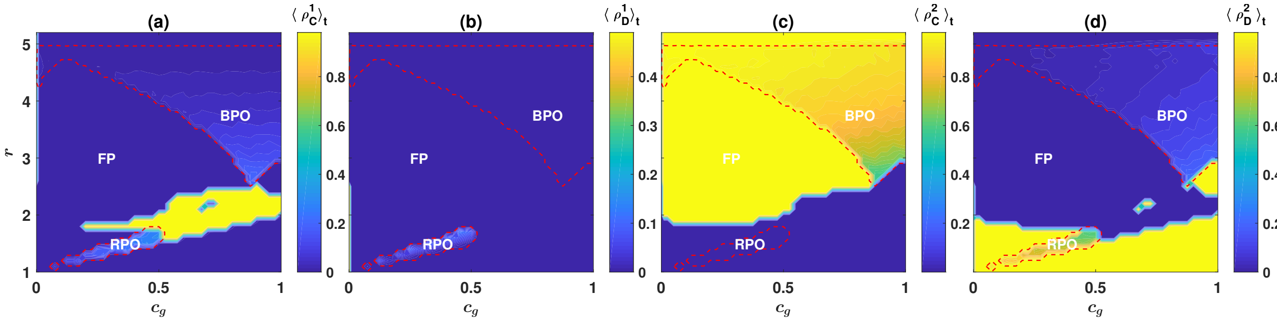

Supplementary Figure.6: The density of different strategies in the $c_g - r$ plane for zero mutation rate. The densities of different strategies in the $c_g - r$ plane are color plotted for zero mutation rate. The dynamics can settle in a fixed point or two different periodic orbits (inside the dashed red lines). For small enhancement factors, the system evolves into a defective fixed point in which all the individuals are non-costly defectors. For larger enhancement factors, cooperation evolves. In the cooperative fixed point, either costly cooperators dominate (high costs) or non-costly cooperators dominate (smaller costs). The dynamics can also settle in a red queen periodic orbits where only cooperators in the costly institution survive and coexist with defectors (for small enhancement factors) or a black queen periodic orbit where cooperators in both institutions survive and show periodic fluctuations in frequency (for large enhancement factors). Parameter values: $\pi_0 = 2$ $g = 5$, $\nu = 2$. The replicator dynamic is solved for 200000 time steps, and time averages are taken over the last 150000 steps.

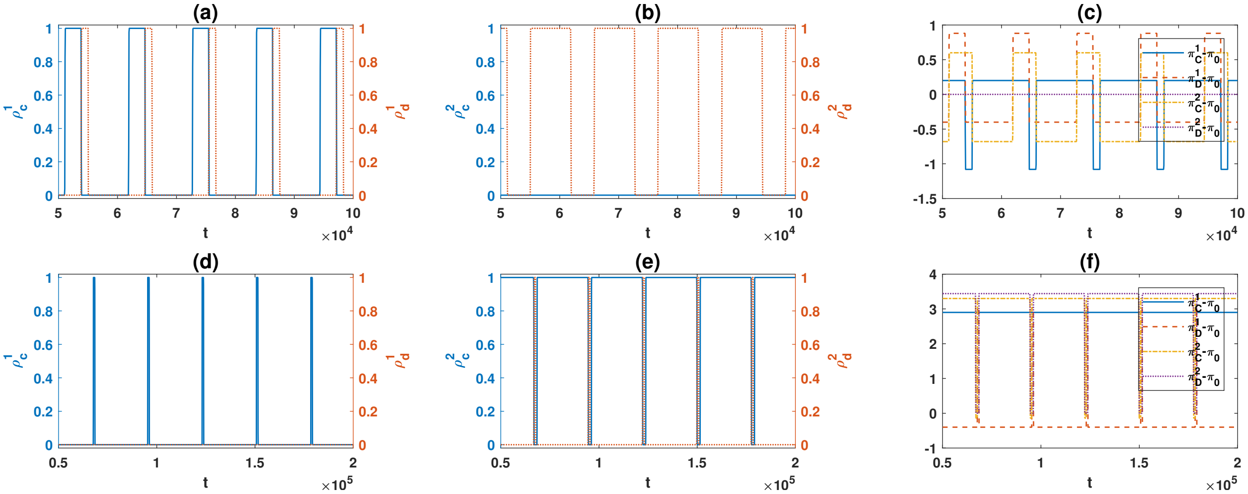

Supplementary Figure.7: The red queen and the black queen orbits under a pure selection dynamic. The density of different strategies (a) and (b), and the game payoffs (c) in the red queen and the black queen, (d) to (f), periodic orbits. In the red queen orbit, cooperators in the costly institution survive and cyclically dominate the population. However, the payoff of the surviving strategies fluctuates around zero. In contrast, cooperators in both institutions survive in the black queen orbit and, together with non-costly defectors, cyclically dominate the population. Besides, the payoff of all the surviving strategies starts to deviate from zero. Parameter values: $g = 5$, $\nu = 0$, $\pi_0 = 2$, and $c_g = 0.4$. In (a) to (c) $r = 1.7$, and in (d) to (f) $r = 4.3$.

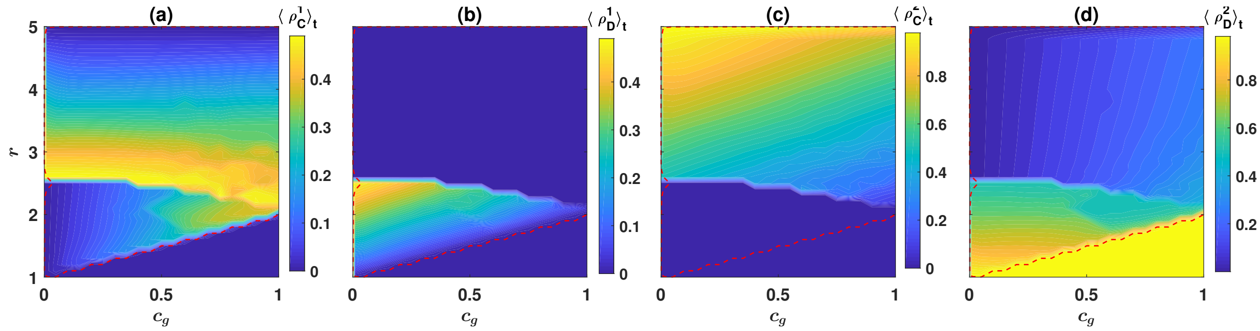

Supplementary Figure.8: The density of different strategies in the $c_g - r$ plane for continuous replicator dynamics. The dynamics can settle in a fixed point or a periodic orbit (the region inside the dashed red line). For small enhancement factors, the red queen periodic orbit, in which cooperation only in the costly institution evolves, occurs, and for large enhancement factors, the black queen periodic orbit, in which cooperation in both the institutions evolves, occurs. Parameter values: $\pi_0 = 2$, $g = 5$, and $\nu = 10^{-8}$. The replicator dynamics are solved for a large enough time for the system to reach stationarity, and the time averages are taken over 30000 time steps.

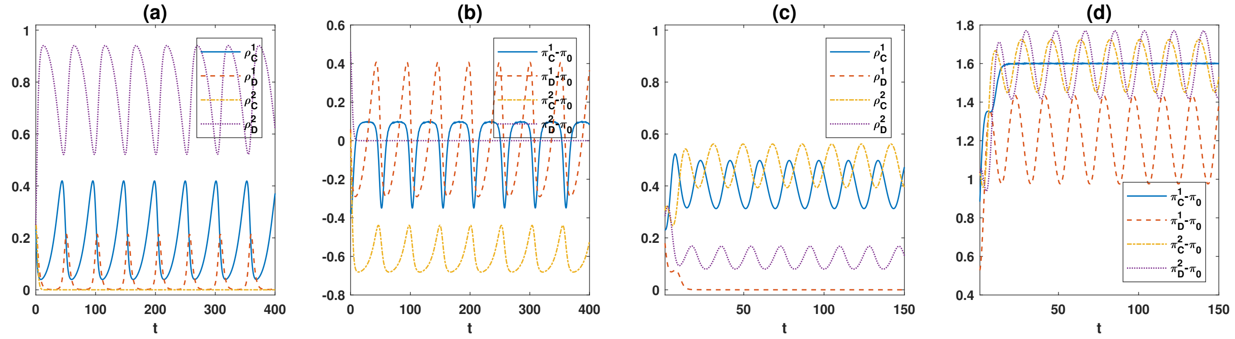

Supplementary Figure.9: Red queen and black queen orbits under the continuous replicator dynamic. The density of different strategies (a) and the game payoffs (b) in the red queen and the black queen, (c) and (d), periodic orbits are plotted. In the red queen orbit, cooperators in the costly institution survive. However, the payoff of the surviving strategies fluctuates around zero, and none dominate others. In contrast, cooperators in both institutions evolve in the black queen orbit, and cooperators of each type suppress defection in their opposite institution. Consequently, the payoff of all the strategies starts to deviate from zero. Furthermore, the frequency of defectors in the costly institution remains very small in the black queen periodic orbit. Parameter values: $g = 5$, $\nu = 10^{-7}$, $\pi_0 = 2$, and $c_g = 0.5$. In (a) and (b) $r = 1.5$, and in (c) and (d) $r = 3$.

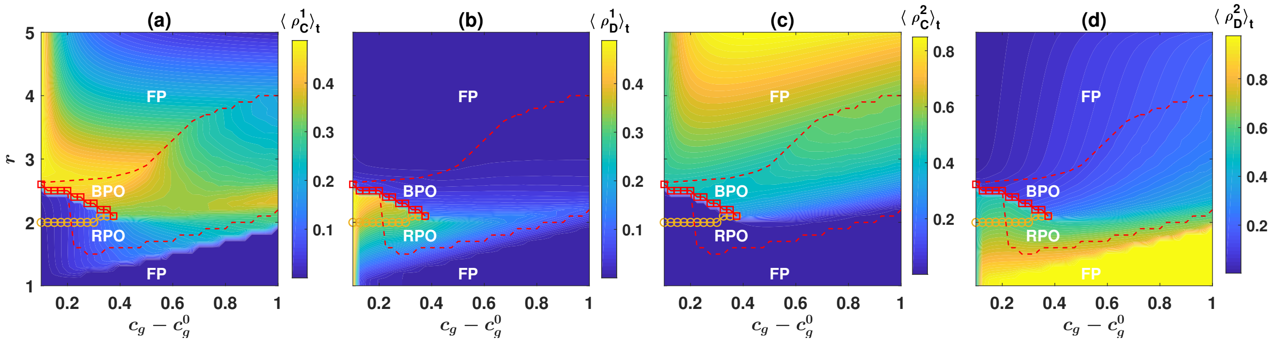

Supplementary Figure.10: The density of different strategies in the $c_g - r$ plane resulting from the replicator dynamics when both institutions are costly. Institution 1 has a participation cost of c_g and institution 2 has a participation cast of $c_g^0 = 0.1$. The densities of different strategies in the $(c_g - c_g^0) - r$ plane are color plotted. The phase diagram of the model is superimposed. The model shows similar behavior to the case when a costly institution and a cost-free institution coexist. For both small and large enhancement factors, r, the dynamics settle in a fixed point, denoted by FP. In between, in the region between the red lines denoted the dynamics settle in the red queen (RPO) or black queen (BPO) periodic orbits. For a small cost, the model is bistable for medium values of r. Orange circles show the lower boundary of the bistable region, above which the black queen periodic orbit becomes stable. The red squares show the upper boundary of the bistable region above which the dynamics settle in the black queen periodic orbit starting from all the initial conditions. Parameter values: $g = 5$, $\nu = 10^{-3}$, and $\pi_0 = 2$. The replicator dynamic is solved for 10000 time steps, and the time averages are taken over the last 2000 steps.

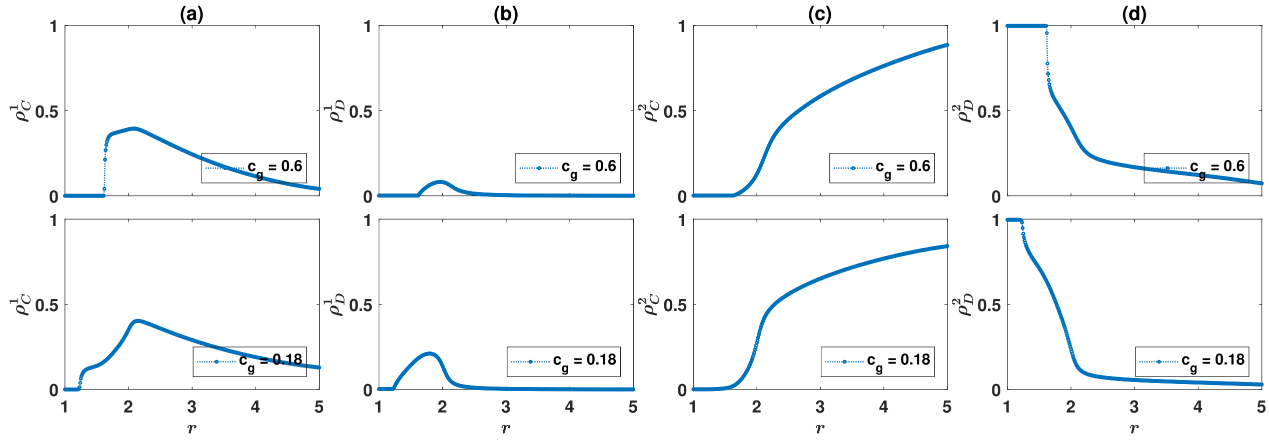

Supplementary Figure.11: Density of different strategies as a function of r for two different values of participation cost in a structured population. The population resides on an 800×800 first nearest neighbor square lattice with von Neumann connectivity and periodic boundaries. The system shows a red queen dynamics in which only cooperators in the costly institution survive in large numbers (for smaller enhancement factors), or a black queen dynamic, where cooperators in both institutions survive and help each other to suppress defection (for larger enhancement factors). Parameter values: $g = 5$, $\nu = 10^{-3}$, and $\pi_0 = 2$. The simulation is performed for 6000 time steps starting from an initial condition in which all the individuals are defectors and prefer one of the two institutions at random. The time averages are taken over the last 2000 steps.

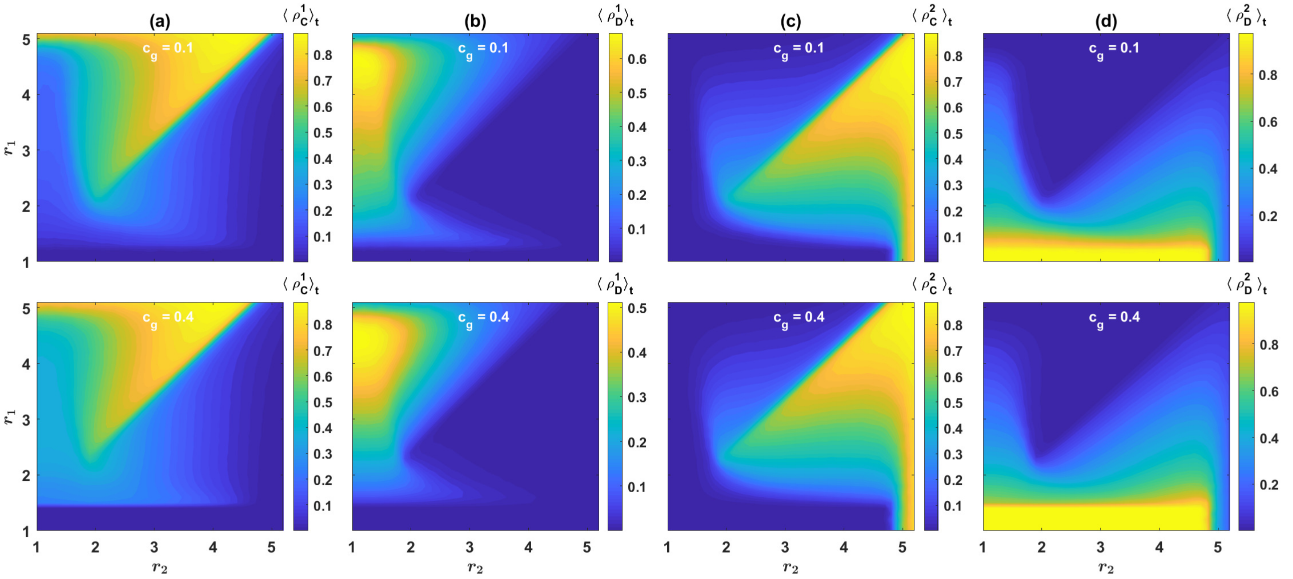

Supplementary Figure.12: The density of different strategies in the $r_1 - r_2$ plane in a structured population. The densities of different strategies in a structured population for two different costs, $c_g = 0.1$ (top) and $c_g = 0.4$ (bottom) are color plotted in the $r_1 - r_2$ plane. The population resides on a 200×200 first nearest neighbor square lattice with von Neumann connectivity and periodic boundaries. Parameter values: $g = 5$, $\nu = 10^{-3}$, and $\pi_0 = 2$. The simulation is performed for 5000 time steps starting from an initial condition in which all the individuals are defectors and prefer one of the two institutes at random. The time average is taken over the last 2000 steps.

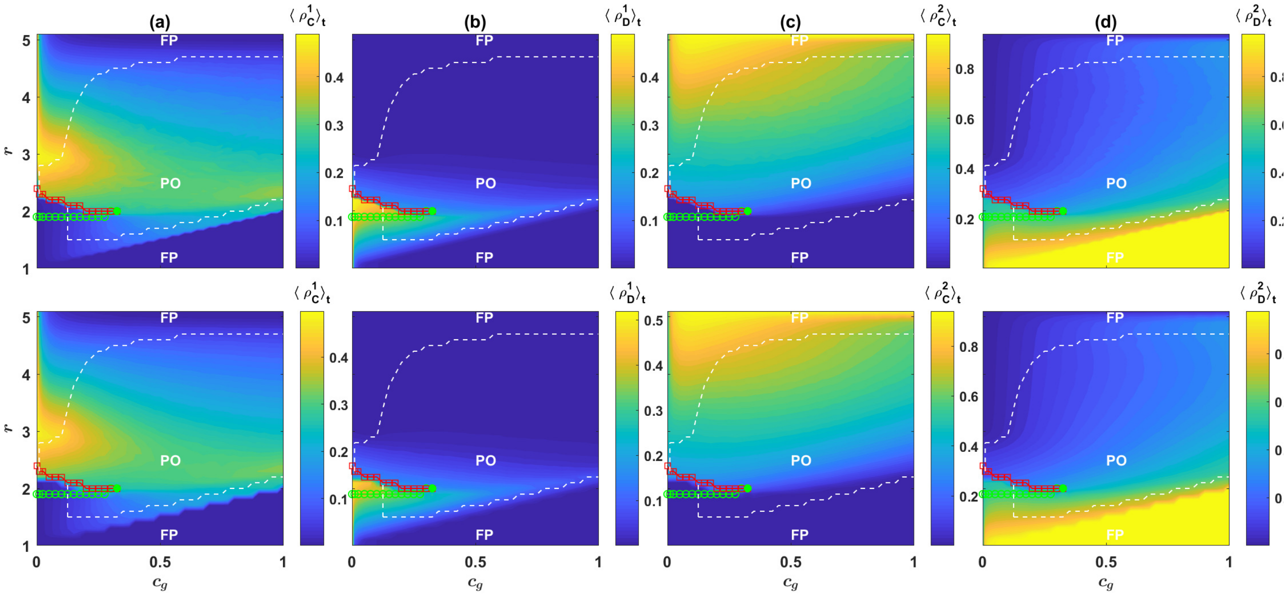

Supplementary Figure.13: The density of different strategies in the $c_g - r$ plane resulting from the replicator dynamics and simulations, in the exponential model. The densities of different strategies in the $c_g - r$ plane are color plotted. The top panel shows the results of the replicator dynamics, and the bottom panel shows the results of simulations. The phase diagram of the model, derived from the replicator dynamics, is superimposed. For both small and large values of enhancement factor, r, the dynamics settle in a fixed point, denoted by FP. In between, the dynamics settle in a periodic orbit, denoted by PO. White lines show the boundary of the cyclic phases. The cyclic phase is composed of a red queen periodic orbit in which cooperation only in the costly institute evolves (for smaller enhancement factors), and a black queen orbit in which cooperation in both institutions evolves (for larger enhancement factors). For a small cost, the model is bistable for medium values of r. Green circles show the lower boundary of the bistable region, above which the black queen periodic orbit becomes stable. The red squares show the upper boundary of the bistable region above which the dynamics settle in the black queen periodic orbit starting from all the initial conditions. The filled green circle shows the point where the transition between the two periodic orbits becomes a continuous transition. Parameter values: $g = 5$, $\nu = 10^{-3}$, and $\beta = 1$. The replicator dynamics are solved for 8000 time steps, and time averages are taken over the last 2000 steps. The simulation is performed in a population of size $N = 10000$ individuals, for 6000 time steps. An average over the last 3000 time steps is taken. The initial condition is a random assignment of the strategies.

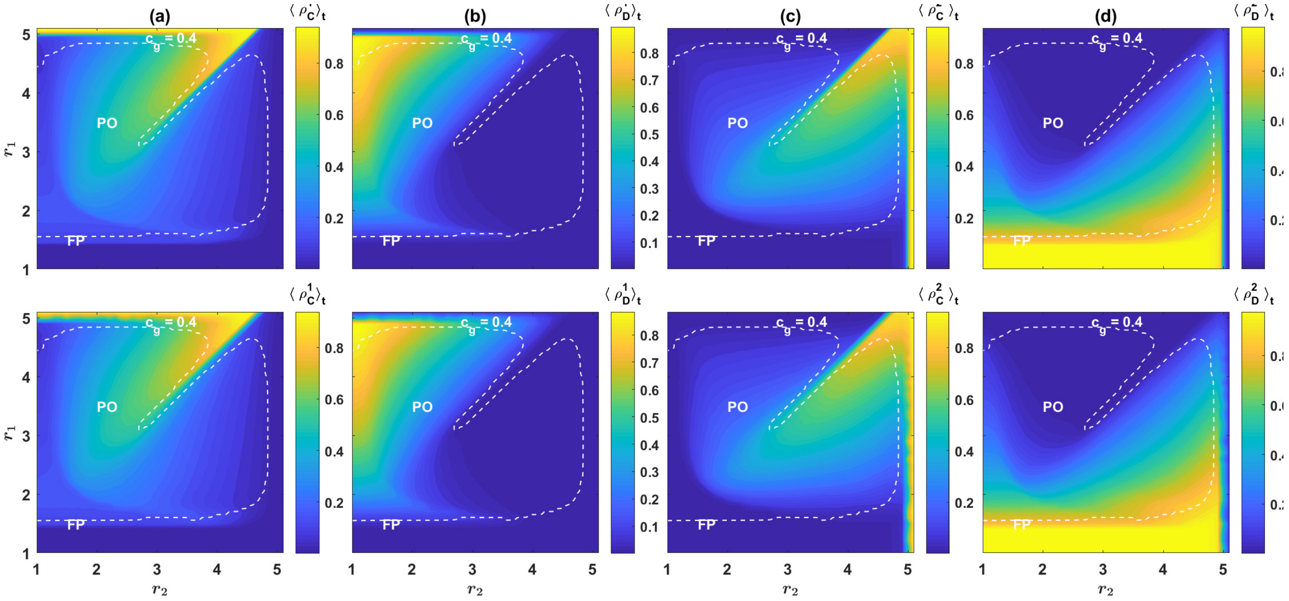

Supplementary Figure.14: The density of different strategies in the $r_1 - r_2$ plane in the exponential model resulting from the replicator dynamics and simulations, for $c_g = 0.4$. The densities of different strategies in the $r_1 - r_2$ plane, resulting from the replicator dynamics (top) and simulations (bottom), are color plotted. The phase diagram of the model, derived from the replicator dynamics, is superimposed. The dynamics settle in a fixed point for both too small and too large enhancement factors. Cyclic fluctuations occur in between. The white dashed line shows the boundary of the region where the dynamics settle in the periodic orbit. The periodic orbit can be a red queen orbit in which cooperation only in the costly institution evolves (small enhancement factors) or a black queen orbit in which cooperation in both institutions evolves (large enhancement factors). Parameter values: $g = 5$, $\nu = 10^{-3}$, and $\beta = 1$. The replicator dynamic is solved for 8000 time steps, and time averages are taken over the last 2000 steps. The simulations are performed for 6000 time steps, and an average over the last 3000 time steps is taken. The initial condition is a uniform initial condition.

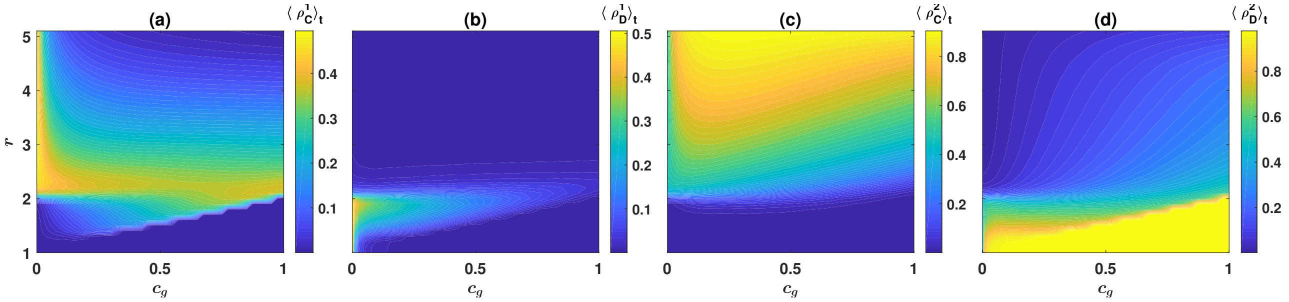

Supplementary Figure.15: The density of different strategies in the $c_g - r$ plane, in a structured population in the exponential model. The densities of different strategies in the $c_g - r$ plane are color plotted. The population resides on a 200×200 first nearest neighbor square lattice with von Neumann connectivity and periodic boundaries. Parameter values: $g = 5$, $\nu = 10^{-3}$, and $\beta = 1$. For too small enhancement factors, defectors prevail. By increasing the enhancement factor, successively, a red queen dynamics in which cooperation only in the costly institution, and a black queen dynamics in which cooperation in both institutions evolves occur. The simulation is performed for 5000 time steps starting from an initial condition in which all the individuals are defectors and prefer one of the two institutions at random. The time average is taken over the last 2000 steps.

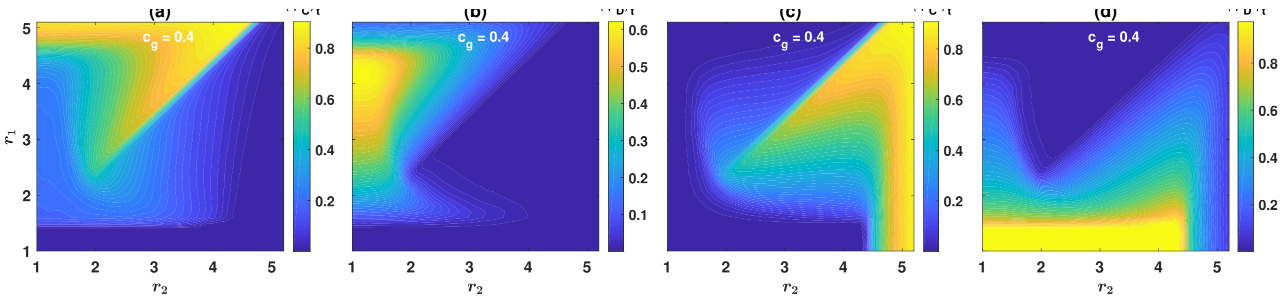

Supplementary Figure.16: The density of different strategies in the $r_1 - r_2$ plane in a structured population in the exponential model. The densities of different strategies in the $r_1 - r_2$ plane are color plotted. The population resides on a 200×200 first nearest neighbor square lattice with von Neumann connectivity and periodic boundaries. Parameter values: $g = 5$, $\nu = 10^{-3}$, $c_g = 0.4$, and $\beta = 1$. The simulation is performed for 5000 time steps starting from an initial condition in which all the individuals are defectors and prefer one of the two institutions at random. The time average is taken over the last 2000 steps.
