# Supplementary material for: Evolution of cooperation in costly institutions exhibits Red Queen and Black Queen dynamics in heterogeneous public goods
Source: Commun Biol. 2021 Nov 29;4:1340. doi: 10.1038/s42003-021-02865-w (PMC8630072; doi:10.1038/s42003-021-02865-w)
Supplement: Supplementary file 3 — Reporting Summary [file 42003_2021_2865_MOESM3_ESM.pdf]

nature research

Corresponding author(s): Mohammad Salahshour

Last updated by author(s): 2021/09/30

Reporting Summary

Nature Research wishes to improve the reproducibility of the work that we publish. This form provides structure for consistency and transparency in reporting. For further information on Nature Research policies, see our Editorial Policies and the Editorial Policy Checklist.

Statistics

For all statistical analyses, confirm that the following items are present in the figure legend, table legend, main text, or Methods section.

n/a	Confirmed	
☒	☐	The exact sample size (*n*) for each experimental group/condition, given as a discrete number and unit of measurement
☒	☐	A statement on whether measurements were taken from distinct samples or whether the same sample was measured repeatedly
☒	☐	The statistical test(s) used AND whether they are one- or two-sided<br>*Only common tests should be described solely by name; describe more complex techniques in the Methods section.*
☒	☐	A description of all covariates tested
☒	☐	A description of any assumptions or corrections, such as tests of normality and adjustment for multiple comparisons
☒	☐	A full description of the statistical parameters including central tendency (e.g. means) or other basic estimates (e.g. regression coefficient) AND variation (e.g. standard deviation) or associated estimates of uncertainty (e.g. confidence intervals)
☒	☐	For null hypothesis testing, the test statistic (e.g. *F*, *t*, *r*) with confidence intervals, effect sizes, degrees of freedom and *P* value noted<br>*Give P values as exact values whenever suitable.*
☒	☐	For Bayesian analysis, information on the choice of priors and Markov chain Monte Carlo settings
☒	☐	For hierarchical and complex designs, identification of the appropriate level for tests and full reporting of outcomes
☒	☐	Estimates of effect sizes (e.g. Cohen's *d*, Pearson's *r*), indicating how they were calculated

Our web collection on statistics for biologists contains articles on many of the points above.

Software and code

Policy information about availability of computer code

Data collection	All the computer codes used in this study were written in MATLAB 2019a or 2017b.
Data analysis	The computer codes used to prepare figures were written in MATLAB 2017b.

For manuscripts utilizing custom algorithms or software that are central to the research but not yet described in published literature, software must be made available to editors and reviewers. We strongly encourage code deposition in a community repository (e.g. GitHub). See the Nature Research guidelines for submitting code & software for further information.

Data

Policy information about availability of data

All manuscripts must include a data availability statement. This statement should provide the following information, where applicable:

- Accession codes, unique identifiers, or web links for publicly available datasets
- A list of figures that have associated raw data
- A description of any restrictions on data availability

The datasets generated during and/or analysed during the current study are available in the ZENODO repository, https://doi.org/10.5281/zenodo.5541182.

Field-specific reporting

Ecological, evolutionary & environmental sciences study design

All studies must disclose on these points even when the disclosure is negative.

Study description	In this computational and mathematical study, a mathematical model is introduced. The dynamics of the model is studied using simulations for two different population structures: A mixed population and a structured population. For a mixed population the replicator-mutator equations are derived and numerically solved using computer codes written in MATLAB. The simulations are performed using computer codes written in MATLAB.
Research sample	No sample was used in this study.
Sampling strategy	No experimental data is involved or collected in this study to require applying a sampling strategy.
Data collection	The data used in this study were generated by computer codes used in simulations and analytical calculations written by the author.
Timing and spatial scale	No experimental data is involved or collected in this study.
Data exclusions	No data were excluded in this study.
Reproducibility	A thorough analysis of the model using simulation and analytical solutions for different parameter values and modeling assumptions ensured the reproducibility of the results.
Randomization	Randomization is not applicable to this study.
Blinding	Blinding is not applicable to this study.

Did the study involve field work? ☐ Yes ☒ No

Reporting for specific materials, systems and methods

We require information from authors about some types of materials, experimental systems and methods used in many studies. Here, indicate whether each material, system or method listed is relevant to your study. If you are not sure if a list item applies to your research, read the appropriate section before selecting a response.

Materials & experimental systems

n/a	Involved in the study	
☒	☐	Antibodies
☒	☐	Eukaryotic cell lines
☒	☐	Palaeontology and archaeology
☒	☐	Animals and other organisms
☒	☐	Human research participants
☒	☐	Clinical data
☒	☐	Dual use research of concern

Methods

n/a	Involved in the study	
☒	☐	ChIP-seq
☒	☐	Flow cytometry
☒	☐	MRI-based neuroimaging
